# Households in HIV Care: Designing an Intervention to Stimulate HIV Competency in Households in South Africa

**DOI:** 10.3389/fpubh.2020.00246

**Published:** 2020-06-30

**Authors:** Caroline Masquillier, Edwin Wouters, Linda Campbell, Anton Delport, Neo Sematlane, Lorraine Tanyaradzwa Dube, Lucia Knight

**Affiliations:** ^1^Department of Sociology, University of Antwerp, Antwerp, Belgium; ^2^School of Public Health, University of the Western Cape, Cape Town, South Africa

**Keywords:** intervention development, 6SQuID framework, HIV, competent households, South Africa, community health workers

## Abstract

Despite the Universal Test and Treat program and widespread antiretroviral treatment rollout, South Africa is still facing HIV prevention and treatment challenges, which are aggravated by human resource shortages in the healthcare sector. Individual- and community-level responses to these HIV-related challenges are increasingly being explored, for example, in community and home-based care. The role of the household as a crucial mediating social level has, however, largely been omitted. This paper outlines the design of an intervention to stimulate the involvement of the household in support for people living with HIV in South Africa. The *6SQuID model* guided the intervention development process in four phases: (1) formative research, theory formulation, and a review of the existing literature, (2) integration of the results from the formative research into the “Positive Communication Process” (P^2^CP model) as a mechanism of change, (3) design of a community-health-worker-led intervention as the way to deliver the change mechanism, and (4) testing and revision of the developed intervention material—called *Sinako*—in a small-scale pilot study. The *Sinako* intervention anticipates that the future of chronic HIV care in resource-constrained settings will need to integrate the patient's household into the fight against HIV.

## Introduction

The human immunodeficiency virus (HIV) epidemic is one of the largest health problems of our time. South Africa is the most severely affected country, with 7.7 million seropositive inhabitants (1). In 2003, the South African government initiated the rollout of an antiretroviral treatment (ART) program in the public health sector (2). This program is now the largest national public health program ever, with 4.8 million people currently receiving ART (1). A 2016 Universal Test and Treat program (UTT) aims to ensure access to ART for all HIV-positive people, irrespective of their CD4 cell count (3). This massive ART scale-up is transforming HIV into a manageable, chronic condition (4)—requiring associated chronic disease management.

The large-scale roll-out of ART is, however, putting an immense burden on the health system, which is already facing severe human resource shortages (5). The inadequate supply and poor retention of health professionals have been defined as one of the most serious obstacles to the sustainable implementation of the treatment plan (6, 7). Sustainable treatment strategies therefore require the mobilization of additional human resources (8). Attention has therefore shifted to the role of community health workers (CHWs) and their capacity to provide chronic disease support (9). Reviews by Tso et al. (10), Wouters (11), and Hall et al. (12) support the conclusion that task shifting to CHWs is one of the key facilitators for linkage to HIV care, ART adherence, and retention in care (10, 12, 13).

In the context of human resource challenges, the South African ART program reaches 62% of the people living with HIV (PLWH). The incidence rates remain high, with 240,000 new HIV infections in 2018, and treatment adherence and retention in care remain challenging—resulting in only 54% of PLWH having suppressed viral loads (VLs) (1, 14). These treatment and prevention challenges highlight the need to develop new sustainable responses—in addition to the involvement of CHWs—to successfully bring an end to the HIV epidemic (14).

A potential source of this much-needed additional support may be found in the PLWH's household. The household, bridging the community (CHWs) and PLWH, is however often overlooked in the current chronic disease care delivery model. PLWH seldom live in isolation, and their home life is generally regarded as the closest and the most basic context for individual development (15). The household forms an “arena of everyday life” where the profound consequences of HIV are experienced [(16), p. 246]. In this regard, the household can be seen as the “first line of health promotion and disease prevention” [(17), p. 3]. “Building health-enabling households” with the capacity to actively stimulate a lifestyle that fosters health offers a promising strategy to support PLWH with adherence and to stimulate preventive practices—thereby responding to current treatment and prevention challenges [(18), p. 4]. The positive role of the household in chronic disease management has been recognized in interventions targeting people with diabetes mellitus (19) and, more recently, in ART adherence interventions (15, 20–22). It must be noted that the household can also form a health-impeding context, where stigma is experienced, making disclosure and treatment adherence more challenging (23). Research is thus required to stimulate households to become a health-enabling context for PLWH, overcoming the challenges of stigma and stimulating positive living.

The aim of this paper is to respond to these research needs by outlining the process of developing an intervention to build a health-enabling household context in which prevention and treatment outcomes are improved for the PLWH and their household members.

## Methods

This intervention development process is part of a larger project —the “Sinako—HIV and Households” study (24). As part of this parent project, a cluster-randomized control trial will test the intervention developed in this paper.

### Research Setting

The HIV epidemic is particularly concerning in the townships where the intervention will be executed in the Cape Metropole, Western Cape Province of South Africa (25). These impoverished areas are densely populated and face severe social and economic challenges, including poor health-related service delivery and a high HIV burden (21.6%) (25).

### The Intervention Development Framework

The “Six Steps in Quality Intervention Development” (*6SQuID*) model guides the intervention development process (26). The 6*SQuID* model has proven to be successful in the development of intervention strategies to improve the health-related behavior of various populations in diverse settings, such as a family-based testing intervention in South Africa (27–29).

This framework for developing social interventions is comprised of six steps: (1) define and understand the problem and its causes, (2) clarify which causal or contextual factors are modifiable and have greatest scope for change, (3) identify how to bring about change: change mechanism, (4) identify how to deliver change mechanism, (5) test and refine the intervention on a small scale, and (6) collect sufficient evidence of effectiveness to justify rigorous evaluation.

## The Intervention Development Process

The first five 6*SQuID* steps are addressed in this paper in four primary research phases: phase 1—formative research, theory formulation, and a systematic review of the existing literature were undertaken, which consequently informed the intervention over the subsequent phases (6*SQuID* steps 1 and 2); phase 2—the findings from phase 1 were then integrated into the “Positive Communication Process” (P^2^CP model) as a mechanism of change (6*SQuID* step 3); phase 3—a community-health-worker-led intervention was designed in order to deliver the change mechanism—based on the work of phases 1 and 2 (6*SQuID step 4*); and phase 4—the developed material is tested and revised in a pilot study on a small scale (6*SQuID* step 5). The results of the *Sinako* cluster-randomized controlled trial will be presented in other publications (6*SQuID* step 6).

### Phase 1: Formative Research, Theory Formulation, and a Review of the Existing Literature

#### Defining the Problem

Despite the largest ART rollout in the country, South Africa is still faced with challenges at every stage of the care continuum. Of the 7.7 million people estimated to be living with HIV in South Africa, 10% do not know their status and are therefore not enrolled into care or on treatment. Furthermore, the incidence rates remain high, with about 240,000 new HIV infections in 2018 (1). Moreover, only 62% of PLWH are on ART (1). Ensuring treatment adherence and retention in care is challenging as 23% of patients enrolled in the South African ART program disengaged from care at least once within a 2-year period (30). In this context, only 54% of PLWH have suppressed VLs (1). This has serious implications for patients as well as for HIV transmission.

#### Theoretical Foundation

To understand the context in which these HIV-related challenges unfold, the research team developed the individual–household–community (IHC) framework, as published in Wouters (11). Departing from the socio-ecological theory, this theoretical framework indicates that the impact of HIV and treatment can be mitigated by interlinked factors at three social levels: (1) the individual, (2) the household, and (3) the community (11). The model integrates a range of sociological concepts into a single robust framework: at the individual level [patient empowerment, stigmatization, and identity management (31–33)], at the household level [communication and health-enabling households (34, 35)], and at the community level [social capital and HIV-competent communities (36, 37)]. These components at the individual, household, and community levels are interlinked: one cannot reduce stigmatization and empower individuals affected by HIV to adhere to treatment and adopt preventive practices without attending to the welfare of the affected households and stimulating health-enabling and HIV-competent communities. Furthermore, empowered household members living positively with HIV and embedded in health-enabling households can be catalysts of preventive practices and positive living within households and communities. However, when this theoretical model was developed, little was known about the role and the function of the crucial intermediate layer, the household (11).

#### Empirically Testing the IHC Theoretical Framework: Quantitative and Qualitative Preparatory Research

As preparation for the trial, the theoretical framework was empirically tested—using Structural Equation Modeling (Mplus)—on existing quantitative data of the randomized controlled trial (RCT) “Free State Effective AIDS Treatment Study” (FEATS) and by qualitative in-depth research (32 audio-recorded in-depth interviews with PLWH and four focus group discussions with 36 CHWs).

The findings of the large-scale quantitative FEATS study demonstrated the crucial role of the household level in chronic HIV treatment, outlined in the IHC framework and published in Wouters et al. (23, 38) and Masquillier et al. (39). The primary research aim of the FEATS study was to investigate the effectiveness of community-based peer adherence support in an RCT in the free state province of South Africa, defining community-based support as peer adherence supporters who had been on ART for at least 12 months and investigating the impact on adherence, measured by the CD4 cell count and self-reported adherence. The results indicated that CHW support had a positive impact on individual ART outcomes in health-enabling households [operationalized using the FACI8 scale (40)], but no impact (or even a negative impact) in health-disabling households, highlighting the potential of intervening at this intermediate household level to optimize CHW support for long-term treatment adherence (23, 38, 39).

However, there was a need to further examine the factors that stimulate a health-enabling environment in households, thereby creating receptivity to the influence of CHW treatment adherence support. By analyzing the transcripts of 32 audio-recorded in-depth interviews with PLWH and four focus group discussions with 36 CHWs—based on the grounded theory approach of Corbin and Strauss (41)—the research team developed the concept of the “HIV-competent household,” as published in Masquillier et al. (42, 43). In this qualitative analysis, the key traits of a health-enabling household for HIV were conceptualized: (1) gain and translate HIV knowledge into health-enhancing behavior, (2) create a social space for dialogue and critical thinking, (3) foster a sense of ownership of the problem and responsibility for its management, (4) build solidarity and a common purpose, and (5) be receptive to outside support (e.g., from a CHW). In an HIV-competent household, sustainable support is provided to the PLWH in their midst, and all household members are encouraged to adopt preventive practices (38, 42).

#### Literature Review

A published systematic literature review was conducted in order to determine how interventions may successfully enhance and develop the traits that render a household health-enabling or HIV competent (44). Following the “Preferred Reporting Items for Systematic Reviews and Meta-Analyses” statement (45), existing household-focused interventions were examined to identify various strategies to improve the management of HIV and the various dimensions of HIV competence. Additionally, the research team sought to explore the intervention mechanisms involved in generating the outcomes of these household-focused interventions (44). The identified HIV-related household-focused interventions were multi-component and multi-dimensional. Most interventions incorporated aspects of information sharing on HIV, improving communication, stimulating social support, and promoting mental health.

The systematic review identified promising mechanisms by which to create HIV-competent households. However, it concluded that studies which actively aim to stimulate HIV competence in households with regards to managing and preventing HIV are lacking. The role of the household as an enabling resource to improve the outcomes of PLWH remains largely untapped; the review concluded that more research on actively improving household HIV competency is required.

#### Identifying Causal and Contextual Factors in Prevention and Treatment Challenges

**Figure 1:**
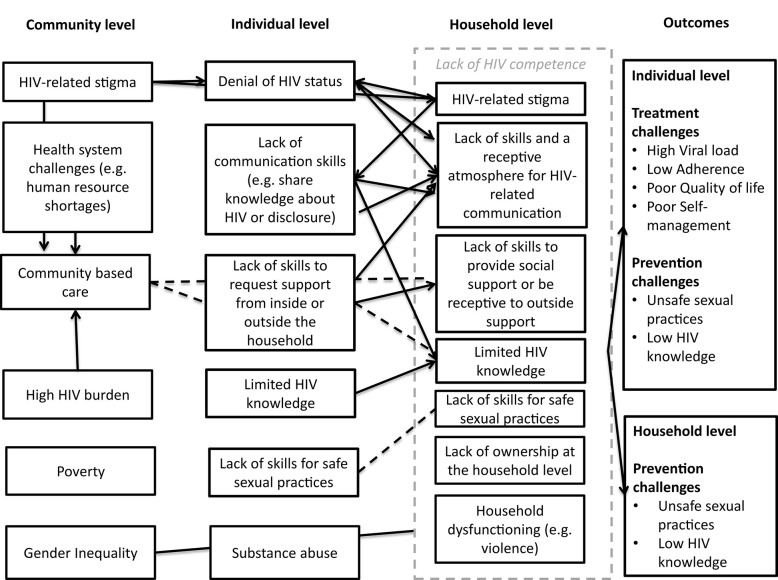
Causal pathways to poor treatment and prevention outcomes.

To finalize the first phase of the intervention development, the research team integrated the results of the formative research into one conceptual model (Figure 1), mapping the causes and contextual pathways that ultimately contribute to poor treatment and prevention outcomes.

In conclusion, various barriers to treatment adherence and preventive behavior exist at the individual, household, and community levels. The individuals affected by HIV (physically, psychologically, and socially) attempt to shape their life with HIV and on ART within their broader social context. As a result, it is not only the responsibility of the individual that should be considered necessary for successful treatment adherence but also the capacity of their household to actively stimulate a lifestyle that fosters health. In this social context, extensive efforts are thus required to increase HIV knowledge, reduce stigma, stimulate HIV testing, improve healthcare-seeking behavior, and encourage safe sexual practices—described in our work as the need for “HIV-competent households” (42).

In responding to the second step of the 6*SQuID*, the team identified which causal or contextual factors were modifiable and provided the greatest potential for positive change—informed by the results of phase 1. These modifiable factors focus on communication at the individual and household levels, such as disclosure and sharing of knowledge. The literature review, the formative qualitative research, and the household functioning measurement of the quantitative RCT all indicated that good household communication was found to be key in the conceptualization of an HIV-competent household. More specifically, by increasing HIV knowledge among household members and by creating a safe space for dialogue about the disease, this might in turn promote preventive practices within the household, such as voluntary HIV testing, and long-term adherence support for the PLWH in their midst.

In the second phase of this intervention development process, these identified modifiable factors form the intervention target.

### Phase 2: Identifying the Change Mechanism: the “Positive Communication Process” (P^2^CP Model)

Armed with the knowledge from phase 1, the research team convened in a series of meetings to integrate the research results (6*SQuID* steps 1 and 2) into a “Positive Communication Process” (P^2^CP model) as a mechanism of change (6*SQuID* step 3). In the P^2^CP process, “positive” refers both to the communication style and the HIV-positive status. The “Positive Communication Process” delineates four steps to build HIV competence at the household level (38, 42, 46).

First, the road to HIV competency commences with the recognition of the reality of HIV by the PLWH themselves (P^2^CP—step 1) as well as by their household members. The household members can only offer social support when the patient has disclosed his or her HIV status (47). HIV disclosure is thus necessary for building household HIV competence. The P^2^CP encourages the patient to disclose to—at least—one household member, preferably one with the power to influence decision-making within the household (P^2^CP—step 2). Then, the patient and the confidant are encouraged to become the change agents who create awareness and openness about the disease in the household once they are equipped with knowledge and the communication skills to drive the move toward household HIV competence (P^2^CP—step 3). Lastly, the change agents will act as “household health advisors” by disseminating the knowledge they have gained on HIV treatment, social support, and prevention to other household members. These constructive dynamics are translated into HIV competence stemming from the positive communication dynamics combined with the increased HIV knowledge at the household level (P^2^CP—step 4).

The end result is a household that forms a health-enabling environment, with effective HIV management by supporting the patient, increasing disclosure and acceptance, and reducing other household members' vulnerability to infection. It is therefore easier for the patient to self-manage their treatment, adhere to ART, and reduce the likelihood of new HIV infection within the household, for instance, by encouraging increased condom use and regular testing.

**Figure 2:**
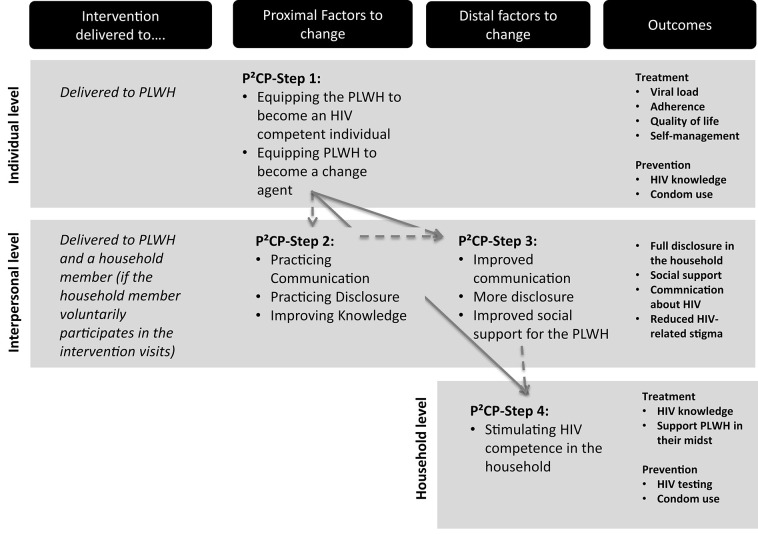
Pathways of change.

The pathways of change—visualizing the causal change processes based upon the above-described theoretical framework—are mapped in Figure 2. The four steps to HIV competence as identified in the P^2^CP model are illustrated in this figure by the arrows.

### Phase 3: Design of a Community-Health-Worker-Delivered Home-Based Intervention

The third phase of the intervention development aimed at translating the P^2^CP model into a CHW-delivered home-based intervention—through a series of workshops (6*SQuID* step 4). Developing the content of the intervention was an iterative process, which builds upon the P^2^CP model—described in phase 2.

#### Intervention Design and Tools

The intervention draws heavily on the results of phases 1 and 2 and thus employs the four steps to HIV competence, as identified in the P^2^CP model. These four P^2^CP steps are addressed in six intervention visits (intervention visits 1–6) and are supplemented with a final visit to create a long-term plan for support (intervention visit 7). On average, a session is envisaged to take about 1 h. The seven intervention visits will be implemented over a course of 6 months, which is similar in length to the standard-of-care visits.

Based on the P^2^CP model, the intervention aims to stimulate positive communication as a facilitator of HIV competence. The PLWH will be invited to bring a household member or sexual partner to each intervention visit. If a new household member/sexual partner joins a session, their knowledge about HIV and ART will be assessed and developed. The household member or sexual partner will then be included in the activities for the remainder of the intervention visits and be invited to join the following sessions as well. If the PLWH does not bring a sexual partner or household member to the session, the CHW will invite the PLWH to do so at the next visit. However, while it is highly encouraged to bring a household member, this is not a prerequisite to continue the intervention.

**Figure 3:**
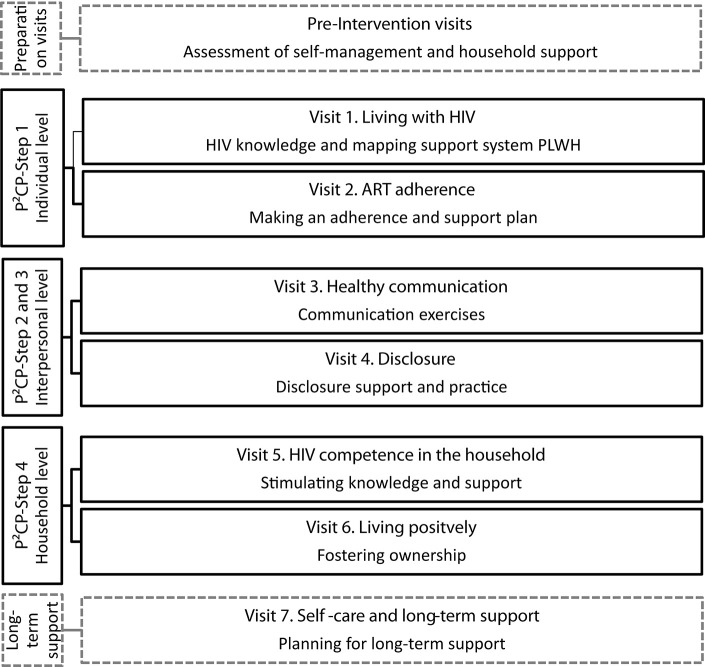
Session overview of the *Sinako* intervention based on the “Positive Communication Process” model.

Figure 3 outlines an overview of the intervention visits. The first two visits focus on individual-level development, stimulating the self-management skills of the patient (P^2^CP—step 1). The first intervention visit begins with building rapport between the PLWH and the CHW using icebreakers and rapport games. Furthermore, the patient's knowledge on HIV and ART is discussed and developed with the support of a visual HIV fact sheet. Next, the CHW and the PLWH discuss how the patient feels about living with HIV and taking ART, using a bulls-eye activity to describe where the patient feels they currently are in relation to where they would like to be. The session ends by completing a genogram to map out the PLWH's relationships with members of their household, presence of sexual partners (within or outside the household), and the nature of those relationships. This is undertaken in order to assess the PLWH's support system. In the second intervention visit, the CHW and the PLWH explore the challenges that the patient experiences with respect to ART adherence. The CHW and the patient work through an adherence plan together, focusing on the following aspects: motivation to take ART, barriers and enablers to adherence, support system, planning for appointments, creation of a medication schedule, reminder strategies, managing missed doses, storing drugs at home, managing side effects, planning for trips, and dealing with substance abuse.

The third and fourth intervention visits focus on the interpersonal level, examining healthy communication and disclosure (P^2^CP—steps 2 and 3). The third intervention visit begins with a discussion about household communication using the genogram as a prompt. The CHW and the patient explore with whom the patient feels comfortable discussing difficult issues and who the PLWH can ask for help from, among other topics. The PLWH subsequently practices positive communication skills, either with the CHW or household member/sexual partner, depending on who is present. For instance, the PLWH learns how to share their HIV-related knowledge with the household and to discuss HIV-related topics such as safe sexual practices and HIV testing. In the fourth intervention visit, the CHW uses the genogram as a prompt to open the topic of disclosure: for example, who has the patient already disclosed to and how was the patient's experience of disclosing in the past? Subsequently, the patient and the CHW discuss the advantages and the disadvantages of disclosing to each person represented in the genogram and to whom the patient may feel comfortable disclosing their status in the future. The CHW then guides the patient in a disclosure role-play exercise to practice disclosing their status to a household member. If the patient does not want to disclose to any household member or sexual partner, the patient is asked whether they are able to identify a person outside the household to whom the patient wants to disclose in order to receive social support.

The fifth and sixth intervention visits focus on the household level, addressing HIV competency in the household (P^2^CP—step 4). The fifth session begins with discussing a new disclosure experience the patient may have had or is still preparing themselves for. The remainder of the visit focuses on HIV competency at the household level. The CHW and the patient discuss how HIV competent their household is; the CHW subsequently supports the patient (and the household member, if present) to become (a) change agent(s) in the household by exploring different scenarios around living with HIV that might occur and by practicing how to respond to these: for example, how to encourage HIV testing among household members or how to encourage others in the household to perform safe sexual practices. The CHW works together with the patient (and household member, if present) to bring together everything they have explored regarding positive communication and HIV knowledge in order to improve HIV competence in the household. The end goal of this step is the creation of an atmosphere in which individuals feel comfortable to have an effective dialogue about the illness and its implications for the life of the patient and the household. In the sixth intervention visit, the CHW facilitates a discussion on how the patient (and household member, if present) can foster ownership of HIV in the household and responsibility for ART adherence, safe sexual practices, and testing of the patient and household members—using a visual “ownership” card game in which various HIV-related household scenarios are presented and actions are discussed. Subsequently, the CHW and the PLWH discuss the things that the patient “cannot change, has control over, and the wisdom to know the difference.” The patient, together with the CHW, then discusses who could support the patient in “things they can control,” such as ART adherence, using the genogram as a prompt. To conclude the session, the genogram is revisited to evaluate how communication and disclosure are developing in the household.

The seventh and last intervention visit completes the intervention by revising the previous sessions and developing a long-term household support plan. The visit focuses on reassessing the (1) self-management skills of the patient using the bulls-eye from session 1 as well as (2) the HIV competence of the household. In revisiting the adherence plan (session 2), the CHW and the patient discuss new challenges that the patient has experienced with respect to adherence and how to tackle these in the future. By making use of the genogram, the CHW and the patient explore who could be of (further) help or support in the household with respect to adherence. A long-term plan of support is developed so that the PLWH is equipped to self-manage his or her treatment within a supportive household environment.

#### Target Population

The participants have to meet the following inclusion criteria for participation in the RCT: a minimum age of 18 years, having commenced ART within 4 weeks of enrolment either for the first time or again (in the case of previous defaulting), having a household member above 18 years old, not being co-infected with tuberculosis at the time of the test, not tested as a result of pregnancy, accessing HIV care and treatment at one of the designated healthcare facilities for this cluster-RCT, and living in the area of this facility.

#### Community Health Workers Delivering the Intervention

The size and the scale of the HIV epidemic compelled South Africa to respond to its health system challenges by developing innovative means of delivering healthcare. The mobilization of CHWs has become an important component of sustainable treatment strategies in the South African health system (48). In line with the IHC framework, the CHWs are at the community level in close proximity to the household level, rendering the cadre of health workers as the most promising and viable way to deliver the change mechanisms. In order to capitalize optimally on the opportunities created by the South African ART program and the UTT program within the context of the limited human resources in the health sector, the CHW will therefore be involved in the delivery of the intervention.

### Phase 4: Testing and Refining the Intervention on a Small Scale

Before the small-scale pilot test, expert stakeholders were invited to provide feedback on the intervention materials. Valuable insight was gained from stakeholders, such as academics with vast experience related to this matter, government officials working in the same context, CHWs familiar with the context, and the implementing NGO, which was then used to develop and revise the intervention. Subsequently, in the last phase, the developed intervention tools were tested and refined on a small scale (6*SQuID* step 5). This process was undertaken with two patients in communities not enrolled in the study. The sessions were recorded and analyzed by the research team. Furthermore, debriefing sessions were held with the CHW delivering the pilot intervention visits and the PLWH receiving the pilot test to assess challenges. The feedback from the piloting has included very few suggested changes to the intervention materials and resources but flagged the importance of allowing for flexibility from the rigid intervention visit scheme by allowing the possibility for “in-between visits” to catch up on any activities that were not finished during the previous visit. Furthermore, at the beginning of each session, the CHW recaps the previous sessions in order to support PLWH with the issues they are working through.

Significant issues were raised by the CHWs around CHW training and comfort with delivering the intervention visits. The training period for CHWs was therefore extended from 6 to 9 days to allow more time to practice the delivery of the intervention, the required communication and delivery skills development, and comfort with the activities and the role. The training and role-playing of the intervention activities formed part of the piloting and feedback process, with the CHWs offering a number of suggestions for changes and adaptations. These mostly related to issues of flow, clarity, and checks for the CHWs.

The strategies developed to prevent and mitigate possible negative effects were discussed with the CHWs during the training workshops. When the CHW or field workers deem necessary, they will provide their patients with contact details for relevant referrals to health or social development government services or community-based or non-governmental organizations experienced in mitigating negative family dynamics and HIV treatment difficulties. Moreover, the CHW will also report back to the research team, which will analyze the feedback in line with the adverse event reporting linked with our ethical approval. The local principal investigator (PI) would make the final call on whether to continue to provide support where appropriate and acceptable to the participants or to withdraw the participants from the study if the research team assessed the study to be in any way negatively impacting the PLWH or their household's circumstances.

## Discussion

This paper has described the four phases of the development process of an intervention aiming to stimulate the potential of PLWH's households to provide a sustainable answer to the HIV prevention and treatment challenges that South Africa is currently facing—more specifically, lack of status awareness, suboptimal treatment adherence and retention, and continuing new infections. The intervention focuses on both the individual and the household levels in order to help the patient to self-manage their ART and to transform their household into an HIV-competent household. As such, the intervention aims to tackle the current treatment and prevention challenges by integrating the household into ART adherence support for the PLWH in their midst and by creating a safe space for dialogue about HIV in order to promote preventive practices. In line with van Rooyen et al. (28), the household is treated as a “social environment (not just as a location for service delivery) through which HIV prevention, treatment, adherence, and support could be achieved” (p. 77).

The *6SQuID* model guided the intervention development process to address the identified steps in four phases. In the first phase, the primary research problem was identified as treatment and prevention challenges. Contextual and causal modifiable factors were explored by drawing on our developed IHC framework, empirical research, and a systematic review. Based on this formative work in the first phase, the “Positive Communication Process” model was developed as the change mechanism. In the third phase, the intervention was designed in several iterations and has been reviewed by experts. The developed intervention is to be delivered by a CHW to a PLWH within seven key counseling sessions within the household to stimulate social support and address aspects of household HIV competency. In the fourth phase of this intervention development, the intervention was piloted on a small scale and refined accordingly.

With regard to using the *6SQuID* intervention development framework, it should be noted that the intervention development was less linear than the *6SQuID* framework suggests but more of an iterative process in which the research team revisited earlier steps during the intervention development process—as also noted by Pringle et al. (29). Nevertheless, the *6SQuID* model proved useful in guiding the research team through all the necessary steps to develop a sound intervention.

However, this study has been subject to several limitations. Although the intervention development has been based on formative quantitative and qualitative research involving PLWHs, little direct consultation with PLWHs has been sustained throughout the intervention development process. Moreover, the reach of our pilot study was limited to only two patients because of time constraints and unexpected changes in the policy landscape which affected employment practices and delayed timelines significantly. The intervention development process has been furthermore challenged by specific policy changes in the local context, namely, shifting responsibilities of NGOs in the area. As Prins et al. (27) noted, collaborating with organizations in the field is very valuable but also challenging. The cooperation with a local NGO made sure that the intervention developed was feasible to implement. The interaction with the stakeholders, like the CHWs and the local NGO, supported the team in tailoring the intervention content to the population and setting. Despite being the best option related to feasibility and sustainability, real life contexts are accompanied by certain challenges, such as the aforementioned health system policy changes.

This study can have both theoretical and practical implications. From a practice and policy perspective, this intervention aims to comprehensively tackle suboptimal adherence and preventive practices by integrating the household in HIV care. For sustainable long-term success, it is vital that PLWH live in households that support and enable the choice of health-enhancing practices, i.e., HIV-competent households. The household constitutes a health-enabling environment, which provides sustainable support to the patient throughout the care continuum—from testing to retention in care—and safe sexual practices become the norm for the PLWH and his or her household members. In an HIV-competent household, a feedback loop might also be created in which other household members are encouraged to be tested and to disclose their status, which is an important step toward a sustainable response to HIV-related challenges. In this intervention, the HIV-competent household concept is pushed beyond the merely theoretical and conceptual level. Building on the team's formative research, the *Sinako* intervention aims to investigate empirically to what extent and in what way HIV-competent households can become sustainable health-enabling contexts that can provide an answer to the prevention and treatment challenges facing South Africa. In order to ensure the sustainability and the feasibility of a potential scale-up of the intervention—when proven successful in the cluster-randomized controlled trial (*6SQuID* step 6)—the intervention arm has only one extra visit in comparison to the standard-of-care arm.

From a theoretical perspective, despite recent emphasis on the importance of social determinants and multi-level interventions, empirical research has lagged behind (49). The strength and the innovation of the current intervention therefore lies in its contribution to the empirical testing of a social ecological model approach (i.e., IHC framework), which theoretically should also optimize the impact of a community intervention on individual health.

## Conclusion

As 7.7 million people are currently infected with HIV and 240,000 people became newly infected in 2018 in South Africa (1), there is an urgent need to explore innovative ways of delivering care in the context of human resource shortages in the health sector. The UNAIDS 2017 report ‘Ending AIDS’ underscored this need, by stating: “providing services closer to where people live and work will be a key factor in ending the AIDS epidemic” [(14), p. 3]. This intervention anticipates that the future of chronic HIV care in resource-constrained settings will need to rely on the integration of the patient's household into the fight against HIV.

## Data Availability Statement

The data that support the findings of this study are available from University of the Free State, but restrictions apply to the availability of these data, which were used under an agreement for the current study, and so are not publicly available. Data are however available from the authors upon reasonable request and with permission of University of the Free State. Requests to access the datasets should be directed to caroline.masquillier@uantwerpen.be.

## Ethics Statement

The studies involving human participants were reviewed and approved by Ethical approval was received from the Ethics Committee of the University of the Western Cape (BM19/4/6) and the Ethical Committee for the Social Sciences and Humanities of the University of Antwerp (SHW_17_64). The patients/participants provided their written informed consent to participate in this study.

## Materials

The developed materials are available from the authors on reasonable request.

## Author Contributions

CM, EW, and LK contributed to the conception and design of the study. CM, EW, LC, AD, NS, LD, and LK contributed to the intervention development. CM wrote the first draft of the manuscript. All the authors contributed to the manuscript revision and read and approved the submitted version.

## Conflict of Interest

The authors declare that the research was conducted in the absence of any commercial or financial relationships that could be construed as a potential conflict of interest.
